# Studies on the Hydrometallurgical Transfer of Lead, Copper, and Iron from Direct-to-Blister Copper Flash Smelting Slag to Solution Using L-Ascorbic Acid

**DOI:** 10.3390/molecules30061365

**Published:** 2025-03-18

**Authors:** Krzysztof Gargul, Arkadiusz Pawlik, Michał Stępień

**Affiliations:** 1 Faculty of Non–Ferrous Metals, AGH University of Krakow, Al. Mickiewicza 30, 30-059 Krakow, Poland; mstepien@agh.edu.pl; 2 Philip Morris Polska, 31-982 Krakow, Poland

**Keywords:** l-ascorbic acid, flash smelting slag, lead recovery, leaching, recycling

## Abstract

This study explored the behavior of lead, copper, and iron during the leaching process of flash smelting slag from direct-to-blister copper flash smelting using l-ascorbic acid solutions. Flash smelting slag is generated in considerable quantities by various copper smelters worldwide. One drawback of the single-stage flash smelting technology for copper concentrates is the production of large quantities of metal-rich by-products. However, through appropriate management of postprocess waste, valuable components such as copper or lead can be recovered. In practice, the slag is typically subjected to decoppering processes involving electric and converter furnaces. The hydrometallurgical process proposed in this study is aimed at replacing high-temperature recovery methods. The primary objective of the experiments was to investigate the effects of variations in specific leaching parameters and the addition of auxiliary substances on the leaching efficiency of lead, copper, and iron. Four parameters were adjusted during the tests: concentration of l-ascorbic acid, liquid-to-solid phase ratio, temperature, and time. An oxidizing agent in the form of perhydrol and citric acid with an oxidant were used as additives. Optimal process conditions were determined to achieve maximum lead leaching efficiency while maintaining relatively low leaching of copper and iron. The experiments indicated that leaching in ascorbic acid solutions resulted in lead extraction efficiencies ranging from approximately 68% to more than 88%, depending on the conditions. Conversely, relatively low leaching efficiencies of iron (4–12%) and copper (0–29%) were observed.

## 1. Introduction

Organic acids such as acetic, citric, and l-ascorbic acids are rarely used substances in industrial processes, particularly in the recycling of nonferrous metals. More commonly used are solutions based on sulfuric, hydrochloric, or other inorganic acids. This is due to the high availability, relatively low cost, and well-established technology for the use of these acids. However, in recent years, the potential use of specific organic reagents in the hydrometallurgical processing of various types of raw materials has gained considerable interest. The use of organic reagents, which are relatively inexpensive and environmentally friendly, may lead to cost reduction in waste disposal or recycling. Examples of such organic acids are acetic, citric, and ascorbic acids. Ascorbic acid, commonly known as vitamin C, exhibits promising properties as an alternative to traditional, often toxic and environmentally unfriendly, metal leaching agents. A literature review allows for several key observations. Studies conducted in recent years have focused on the use of ascorbic acid solutions in the extraction of metals from both primary and secondary sources. In particular, numerous analyses were conducted on the recovery of cobalt, nickel, manganese, and lithium from spent lithium-ion batteries [1,2,3,4,5], extraction of rare earth metals from neodymium magnets [6,7,8], or other primary and secondary sources [9,10,11]. Attempts have also been made to use ascorbic acid for copper or zinc recovery from flotation tailings [12,13]. In addition to the aforementioned studies, some studies have also described the leaching of copper, silver, and gold from printed circuit boards [14,15], indium and other nonferrous metals from LCD monitors [16,17], recovery of vanadium and nickel from heavy oil fly ash [18], and dissolution of copper, cobalt, manganese, and nickel from oceanic nodules [19,20]. There are also studies describing the removal of lead and other toxic metals from heavily contaminated soil using various organic compounds, including ascorbic acid [21,22]; leaching of lead compounds from waste generated during the processing of used lead-acid batteries [23]; extraction of Cd, Cu, Pb, and other heavy metals from green tea [24]; or recovery of uranium from monazite [25]. The effectiveness of metal leaching depends on various factors such as the concentration of ascorbic acid, temperature, time, and particle size of the processed material. Some studies reported the possibility of process optimization to increase efficiency or achieve higher selectivity toward specific metals. Compared with traditional leaching solutions, ascorbic acid is safer for the environment and human health. It is biodegradable and nontoxic, making it a more attractive option from the perspective of sustainable development. A literature review indicates almost no research on the use of ascorbic acid solutions for the recovery of valuable metals from secondary raw materials of the metallurgical industry such as slags, dusts, sludges, scale, and others.

This study focused on the application of ascorbic acid solutions for leaching lead, copper, and iron from slag obtained from the one-stage process of smelting copper concentrates to blister copper [26]. Copper production from sulfide concentrates involves two main stages: smelting to produce matte and conversion to matte. In the continuous direct-to-blister copper smelting process, these two stages are combined into one. The main advantages of this process are that a single, continuous, SO_2_-rich gas stream can be obtained and energy consumption as well as capital and operating costs are minimized. The disadvantages of the process are that about 25% of the copper entering the smelting furnace ends up in the slag and the cost of pyrometallurgical extraction of copper from slag is high. This process is based on the reduction of cuprous oxide and other metals, mainly lead and iron, in the liquid state in an electric furnace in the presence of coke and technological additives. After reduction, a Cu–Pb–Fe alloy is obtained, which is further processed in a converter to remove impurities [27,28]. Hydrometallurgical treatment of the slag could be an alternative route to the currently used method of processing. This study aimed to examine the influence of various leaching parameters such as process time, concentration of ascorbic acid, l/s parameter (volume of leaching solution to mass of solid phase), and temperature on the leaching efficiency of Pb, Cu, and Fe.

## 2. Results and Discussion

Leaching tests of flash smelting slag were conducted according to the procedure described previously and were divided into two parts:

- Investigation of the behavior of Cu, Pb, and Fe under leaching conditions with l-ascorbic acid solutions—optimization of the process for maximum lead leaching efficiency.
- Investigation of the influence of auxiliary substances such as perhydrol or citric acid on the leaching process of the slag.

### 2.1. Optimization of the Leaching of Flash Smelting Slag Using L-Ascorbic Acid Solutions

In this section, the leaching efficiency of lead, copper, and iron from flash smelting slag was determined using l-ascorbic acid solutions. The study involved observation of the influence of various leaching parameters such as concentration, liquid-to-solid phase ratio (l/s), and temperature on the leaching efficiency of lead, copper, and iron. The leaching parameters were adjusted to increase the leaching efficiency of lead while minimizing the leaching efficiency of iron and copper.

#### 2.1.1. Effect of L-Ascorbic Acid Concentration on the Leaching of Lead, Copper, and Iron

**Figure 1:**
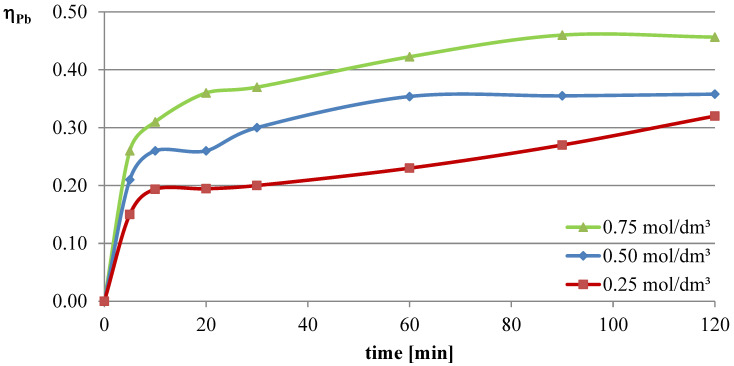
Leaching efficiency of lead from flash smelting slag using l-ascorbic acid solutions.

**Figure 2:**
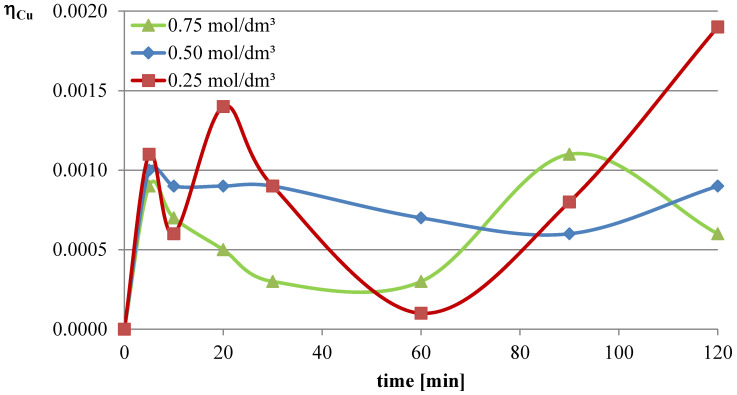
Leaching efficiency of copper from flash smelting slag using l-ascorbic acid solutions.

**Figure 3:**
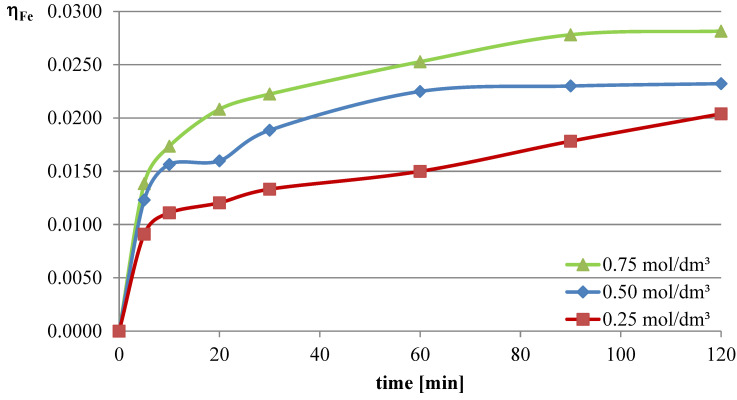
Leaching efficiency of iron from flash smelting slag using l-ascorbic acid solutions.

In the first series of leaching experiments, investigations were conducted under conditions where all the leaching parameters were fixed, except for the concentration of l-ascorbic acid. The concentrations of acid in individual trials were 0.25, 0.5, and 0.75 mol/dm^3^. The remaining leaching parameters had the following values: T = 50 °C, l/s = 10. In all the series of experiments presented in this study, a constant stirring speed of 250 rpm was set. This speed ensures the highest possible leaching efficiencies for specified geometrical dimensions of the beaker, stirrer, and filling degree of the leaching solution. The obtained results are presented in the leaching efficiency vs. time plots (Figure 1, Figure 2 and Figure 3 for lead, copper, and iron) and the pH values of the solution (Table 1).

The obtained results clearly indicate that the leaching efficiency of lead from flash smelting slag increases with the increase in the concentration of l-ascorbic acid. Under the conditions of the first measurement series, it was possible to leach approximately 46% of the lead in the initial slag sample after 90 min of experimentation when using an acid solution at a concentration of 0.75 mol/dm^3^. For iron, a directly proportional relationship between leaching efficiency and the concentration of the leaching solution is also observed. However, in the case of this element, the maximum leaching efficiency of this measurement series was only approximately 2.8%. Through leaching of copper using ascorbic acid, trace amounts of this element in the solution can be obtained. The maximum leaching efficiency of copper measured in this section was just below 0.19%. Therefore, it can be concluded that l-ascorbic acid solution exhibits relatively high selectivity toward the three metals analyzed in the study.

#### 2.1.2. The Effect of the l/s Parameter on the Leaching of Lead, Copper, and Iron

**Figure 4:**
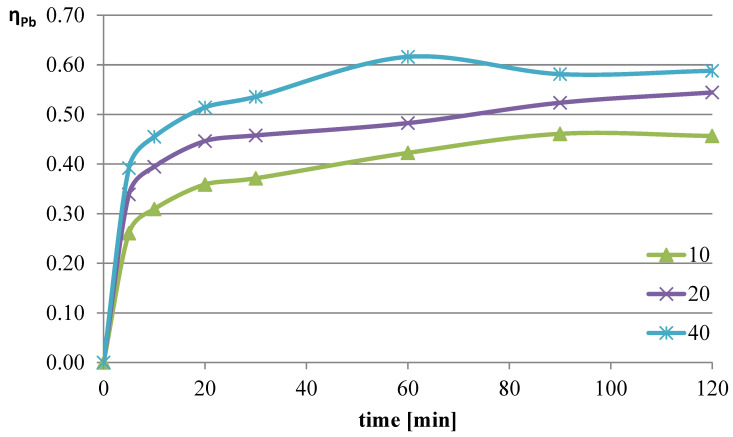
Leaching efficiency of lead at different values of l/s.

**Figure 5:**
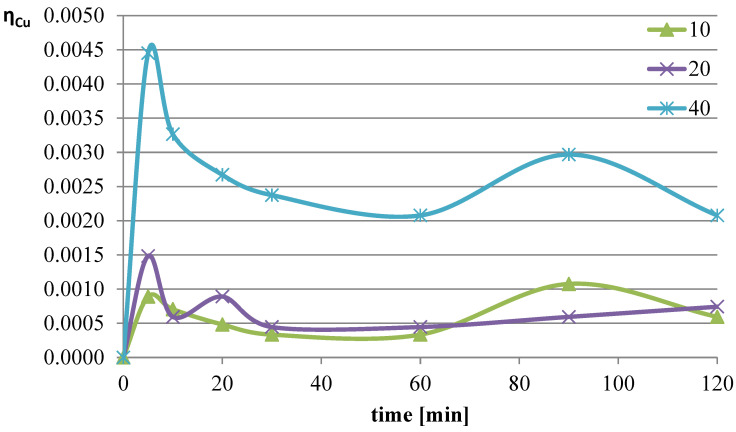
Leaching efficiency of copper at different l/s values.

**Figure 6:**
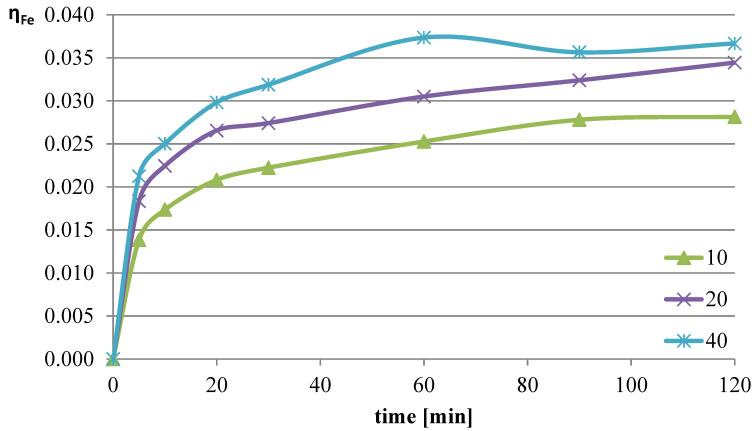
Leaching efficiency of iron at different l/s values.

The next parameter investigated was the ratio of the volume of the liquid phase (leaching solution) to the mass of the solid phase (mass of slag sample). Increasing the l/s ratio results in a greater amount of reacting substance relative to the mass of metals contained in the leached slag. The experiments were conducted at a temperature of 50 °C, and the l-ascorbic acid concentration was 0.75 mol/dm^3^. The l/s parameter in individual trials was set to 10, 20, and 40. The graphs in Figure 4, Figure 5 and Figure 6 show the change in the leaching efficiency of lead, copper, and iron as a function of the process time. The pH values of the solution during the experiments are presented in Table 2.

Studies on the influence of the change in the l/s ratio have demonstrated that for all elements, increasing this parameter enhances the leaching efficiency of the analyzed components of the slag. The maximum leaching efficiency of lead, reaching almost 62%, was achieved after 60 min of the process at l/s = 40. Under the analyzed conditions, copper practically did not dissolve into the solution, and the leaching efficiency of iron reached a maximum of about 3.7%. Because maximizing the leaching efficiency of lead was the priority in the planned studies, it was therefore concluded that in further tests, the l/s ratio of 40 would provide the highest degree of lead transfer from flash smelting slag to the leaching solution.

#### 2.1.3. Effect of the Temperature of the L-Ascorbic Acid Solution on the Leaching of Lead, Copper, and Iron

**Figure 7:**
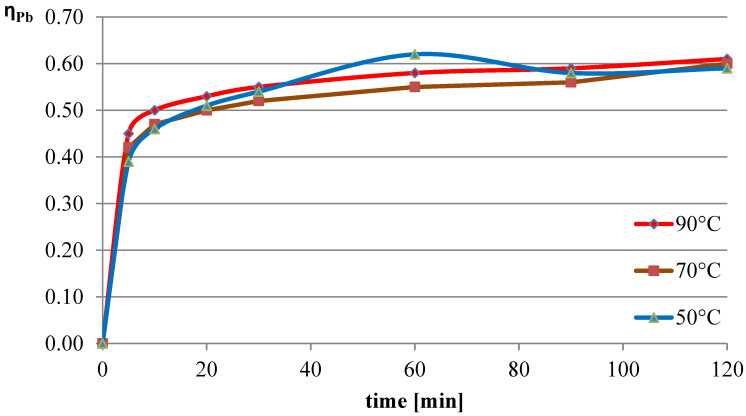
Leaching efficiency of lead at different process temperatures.

**Figure 8:**
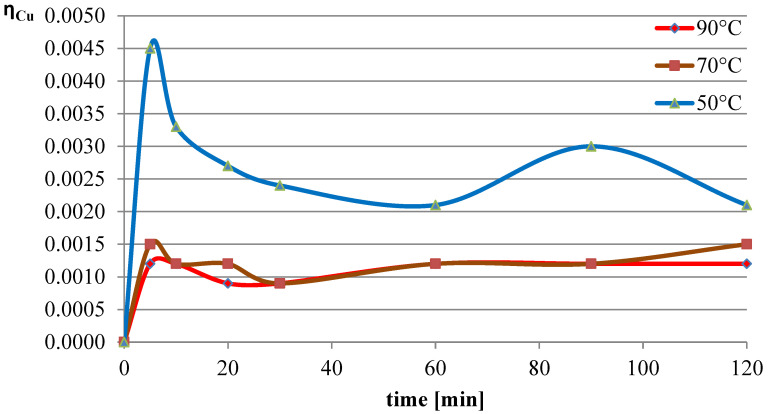
Leaching efficiency of copper at different process temperatures.

**Figure 9:**
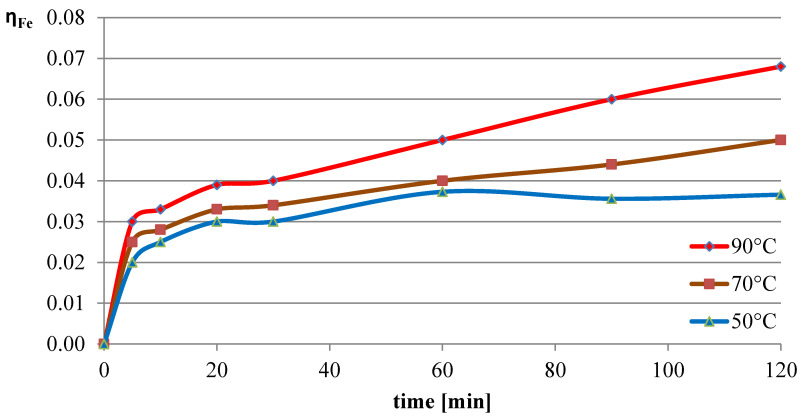
Leaching efficiency of iron at different process temperatures.

The next variable leaching parameter was temperature, which can affect the reaction rate and process efficiency. Investigations were conducted for three selected temperatures: 50 °C, 70 °C, and 90 °C. The experiments in this section were conducted with the l-ascorbic acid concentration set to 0.75 mol/dm^3^ and the l/s parameter to 40. The results of the experiments in the graphical form are shown in Figure 7, Figure 8 and Figure 9. The pH values of the solution over the course of the process for the three analyzed temperatures are presented in Table 3.

The obtained results indicate that the process temperature does not substantially affect the leaching of lead. The leaching efficiencies obtained in all three trials were similar to each other, with the highest recorded after 60 min of the process at the lowest temperature. Copper, under the analyzed conditions, practically did not dissolve into the solution. In the case of iron, a clear positive correlation was observed between leaching efficiency and process temperature. Under the conditions of maximum leaching time and highest temperature, a process efficiency of just below 7% was achieved.

The study conducted in this part of the work indicates that the optimal conditions for leaching flash smelting slag using l-ascorbic acid solution are 50 °C, with the acid concentration set to 0.75 mol/dm^3^ and the l/s ratio to 40. This resulted in a highly selective leaching process, as after 60 min of testing, a lead leaching efficiency of 62% was achieved, with the copper and iron leaching efficiencies being 0.2% and 3.7%, respectively.

### 2.2. Leaching of Flash Smelting Slag with L-Ascorbic Acid Solutions with Auxiliary Substances

In the next section, the leaching solution was supplemented with H_2_O_2_ additive and conducted under conditions that allow for achieving the maximum lead leaching efficiency from slag. The behavior of lead, copper, and iron was determined by adding various amounts of H_2_O_2_ to the leaching solution. In the final stage of the study, the influence of the addition of hydrogen peroxide and citric acid to the ascorbic acid solution on the leaching efficiencies of the analyzed elements was determined.

#### 2.2.1. Leaching of Lead, Copper, and Iron in L-Ascorbic Acid Solution with the Addition of Hydrogen Peroxide

This series of tests aimed to determine the effect of the addition of an oxidizing agent to the l-ascorbic acid solution on the leaching process of lead, copper, and iron. In the experiments, hydrogen peroxide, a 30% solution of H_2_O_2_, was used. The oxidizing agent was added in portions as follows: the amount of oxidizing agent was divided into four equal parts and added to the leaching solution. The first portion was added at the start of the measurement, and the subsequent three portions were added every 30 min.

**Figure 10:**
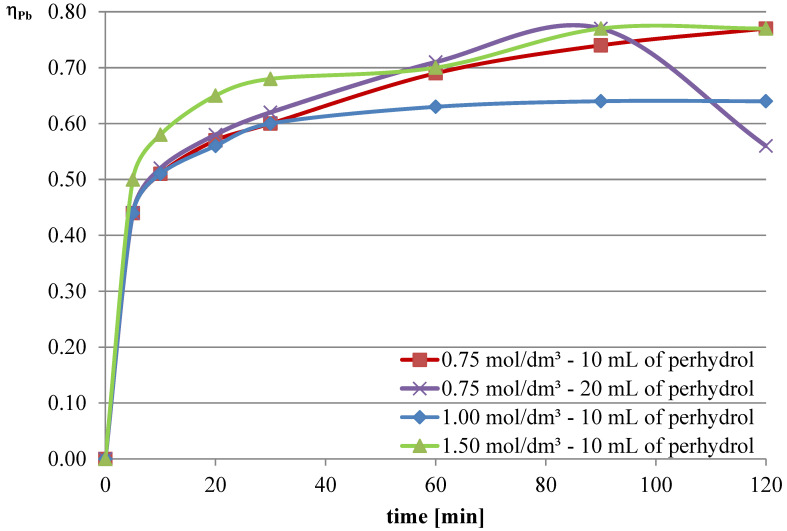
Leaching efficiency of lead for the leaching mixture with the addition of H_2_O_2_.

**Figure 11:**
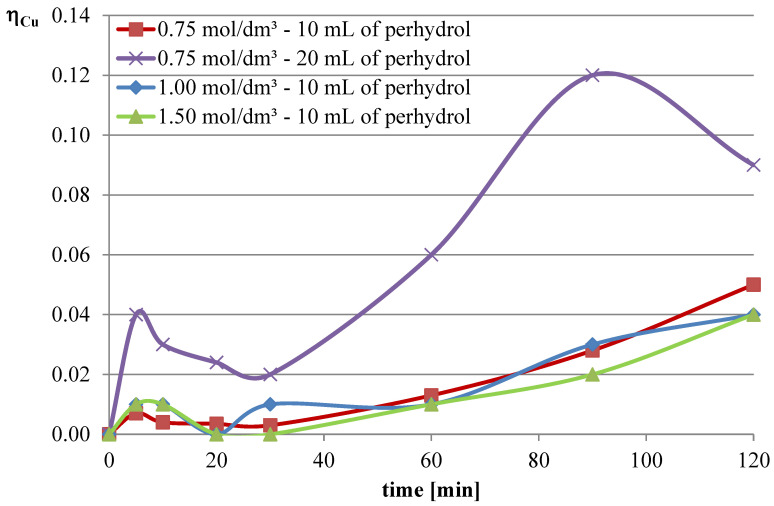
Leaching efficiency of copper for the leaching mixture with the addition of H_2_O_2_.

**Figure 12:**
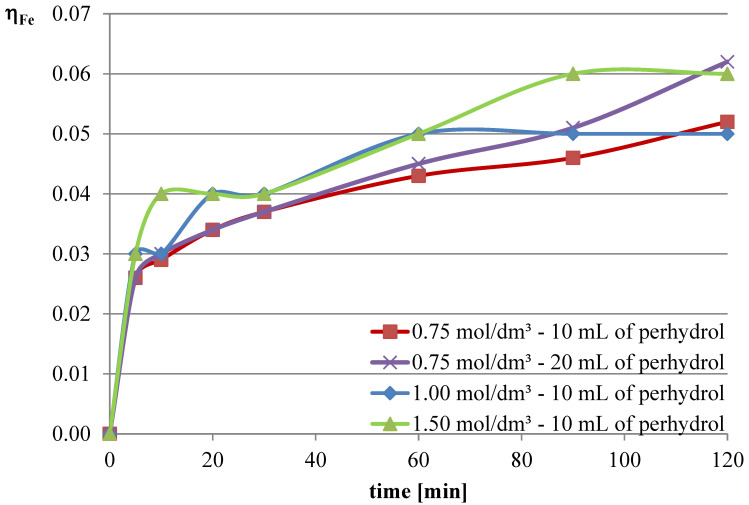
Leaching efficiency of iron for the leaching mixture with the addition of H_2_O_2_.

Experiments with the addition of the oxidizing agent were conducted in two variants. The first two series were conducted with an l-ascorbic acid concentration of 0.75 mol/dm^3^ with the addition of 10 and 20 mL of hydrogen peroxide. Then, it was decided to deviate from the previously established concentration of l-ascorbic acid and conduct experiments under conditions of higher acid concentration. Therefore, in the next two series, l-ascorbic acid solutions with concentrations of 1 and 1.5 mol/dm^3^ were utilized, with the addition of 10 mL of hydrogen peroxide in each case. The obtained results are depicted in Figure 10, Figure 11 and Figure 12, and the pH values of the solutions during the conducted experiments are included in Table 4.

The obtained results indicate that the addition of the oxidizing agent intensifies the leaching processes. In the case of lead, a maximum leaching efficiency of approximately 77% was achieved, representing an increase of over 24% compared with trials without hydrogen peroxide. Maximum efficiencies were obtained for l-ascorbic acid concentrations of 0.75 mol/dm^3^ (10 mL of hydrogen peroxide, 120 min, and 20 mL of hydrogen peroxide, 90 min) and 1.5 mol/dm^3^ (10 mL of hydrogen peroxide, 90–120 min). With the addition of hydrogen peroxide, the leaching efficiency of copper from flash smelting slag also increased. The highest values of this parameter were achieved when l-ascorbic acid solution with a concentration of 0.75 mol/dm^3^ was used with the addition of 20 mL of hydrogen peroxide. The maximum leaching efficiency of copper was achieved after 90 min and reached 12%. There were also some increases in the leaching efficiency of iron when using the oxidizing agent. The use of l-ascorbic acid solution with a concentration of 0.75 mol/dm^3^ with the addition of 20 mL of hydrogen peroxide resulted in the dissolution of 6.2% of the iron content in the slag into the solution.

#### 2.2.2. Influence of Citric Acid Addition to Ascorbic Acid on the Leaching of Lead, Copper, and Iron

**Figure 13:**
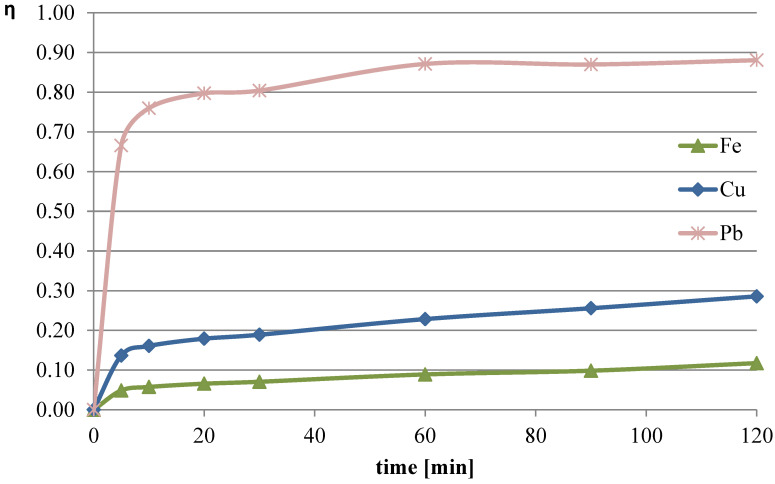
Leaching efficiency of lead, copper, and iron for the leaching mixture (ascorbic and citric acids) with the addition of hydrogen peroxide.

In the last experimental series, it was decided to use a leaching solution consisting of ascorbic acid with the addition of hydrogen peroxide and citric acid (99.5% Sigma-Aldrich). From the literature [29], it is known that leaching of flash smelting slag can be successfully performed using citric acid. However, the combination of citric and ascorbic acids for the leaching of this material and the behavior of individual metals have not been explored. In this trial, a mixture of acids (each at 0.75 mol/dm^3^) with the addition of 10 mL of hydrogen peroxide (four portions added at 0, 30, 60, and 90 min) was used. The process temperature was 50 °C, and the l/s parameter was set to 40. The leaching efficiencies obtained in this trial are shown in Figure 13. The pH values of the solution during the process are shown in Table 5.

The use of this type of leaching solution resulted in the highest recorded leaching efficiency of lead (over 88%). Notably, after just 60 min of the process, approximately 87% of the lead content in the slag had already leached into the solution. The copper leaching efficiency substantially increased compared with the previous measurement series, reaching a maximum of 29% in this trial. The leaching efficiency of iron after 120 min of the process was 12%, which was about twice as much as in the trial using only ascorbic acid with the addition of hydrogen peroxide.

### 2.3. Analysis of the Selected Leaching Products

After conducting all the laboratory tests, the solid residues and the solution obtained after leaching in the following trials with the highest lead leaching efficiency were analyzed:

(A) leaching of slag using ascorbic acid in the presence of an oxidizing agent (l-ascorbic acid concentration of 0.75 mol/dm^3^, 10 mL of hydrogen peroxide, 50 °C, 120 min);
(B) leaching of slag using a mixture of ascorbic acid and citric acid in the presence of an oxidizing agent.

**Figure 14:**
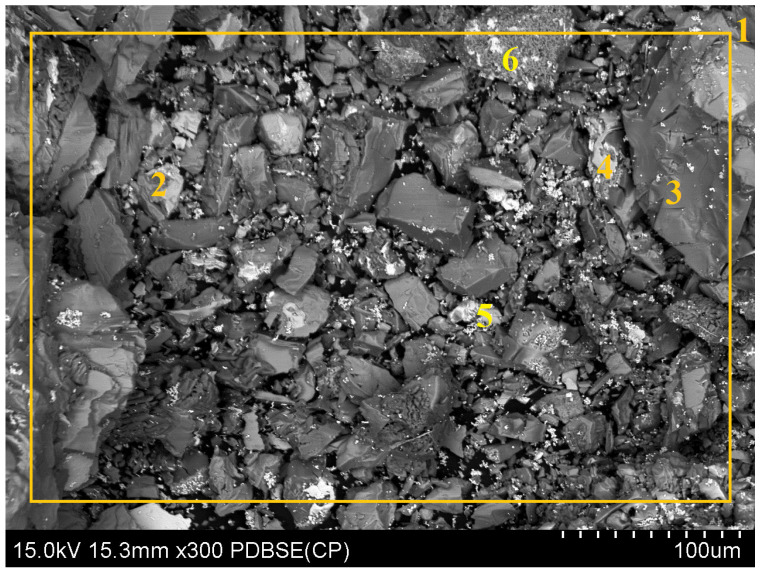
SEM of the solid phase after leaching using ascorbic acid in the presence of an oxidizing agent along with the observed field (1) and characteristic points (2–6).

**Figure 15:**
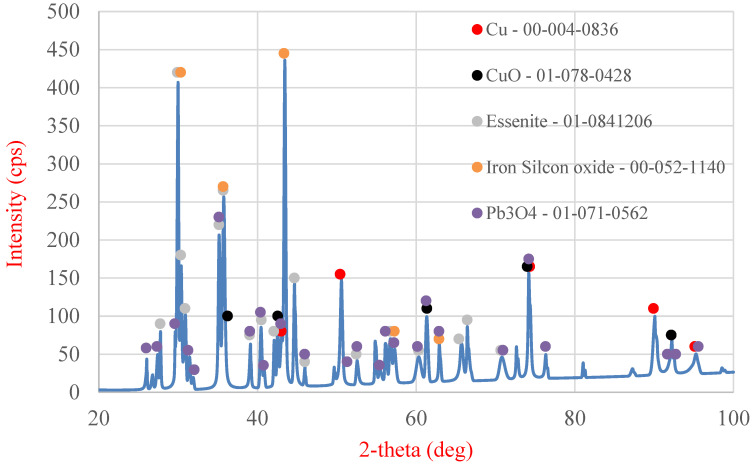
XRD analysis of the sediment after leaching using ascorbic acid in the presence of an oxidizing agent.

The solid samples from variant “A” were subjected to SEM analysis (Figure 14 and Table 6) and XRD analysis (Figure 15), whereas the liquid phase was analyzed using ICP-OES (Table 7). Table 6 shows the area and point measurements of the elemental content. Extremely low lead concentrations are observed in the area measurement (0.3%). The leaching efficiency calculated based on the EDS analysis results (92%) is considerably higher than that calculated based on the results obtained from MP-AES spectrometry (77%). The XRD analysis results are shown in Figure 15, indicating a lower number of phases compared with the original sample. The analysis revealed the absence of the PbO phase, which was removed from the slag during leaching. The ICP-OES analysis (Table 7) revealed similar concentrations of Cu, Pb, and Fe to those shown during analysis on the MP-AES spectrometer. The lead leaching efficiency calculated based on the lead concentration in the leachate (ICP-OES) was 79.3%. In addition, other elements such as calcium, aluminum, sodium, and silicon are present in the postreaction solution.

**Figure 16:**
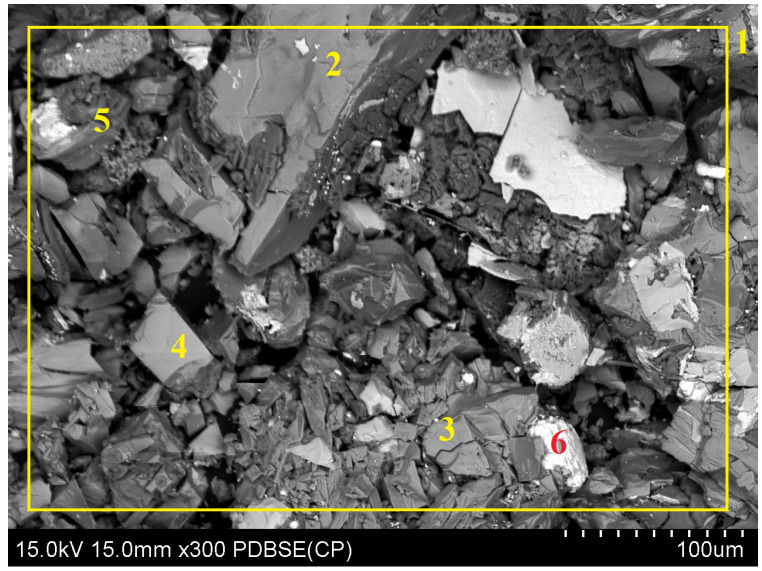
SEM of the solid phase after leaching using a mixture of ascorbic acid and citric acid in the presence of an oxidizing agent along with the observed field (1) and characteristic points (2–6).

**Figure 17:**
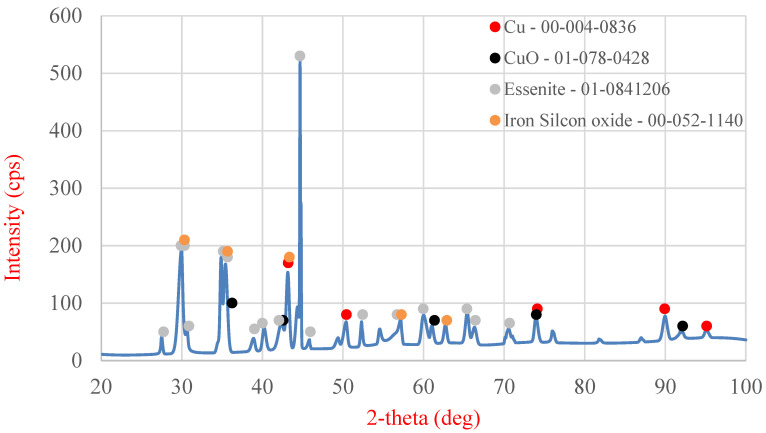
XRD of the sediment after leaching using a mixture of ascorbic acid and citric acid in the presence of an oxidizing agent.

Samples from leaching in variant “B” include the residue after leaching conducted using ascorbic and citric acids in the presence of an oxidizer as well as the leachate. Analogously to variant “A”, SEM analysis (Figure 16 and Table 8) and XRD analysis (Figure 17) of the solid sample were conducted, and the liquid phase was analyzed using ICP-OES (Table 9). The analysis of the results depicted in Table 8 revealed that lead is not present in the analyzed measurement area. However, trace amounts of this element are found on some grains of the obtained material. These data could suggest that the leaching process of lead proceeded with an efficiency of almost 100%. XRD analysis did not reveal any lead-bearing phases in the analyzed material. Calculation of the results obtained from the ICP-OES analysis of the leachate indicated that the leaching efficiency of lead in this variant was 90.6%, which was close to the value obtained from the MP-AES analysis (88.1%).

## 3. Interpretation of Results

The slag under hydrometallurgical processing in this study has many components. The most interesting ones are lead (2.98%), copper (13.48%), and iron (11.67%). XRD analysis revealed the presence of six primary phases constituting this slag, namely, lead-bearing phases such as PbO and Pb_3_O_4_, copper-containing phases such as metallic copper and copper(II) oxide, and two other phases containing iron and other elements. Fine-grained slag (below 0.5 mm) was used for the study, where no dominant grain fraction can be identified.

The leaching studies of slag using ascorbic acid solutions aimed to determine the leaching efficiency of lead, copper, and iron over time depending on three leaching process parameters: concentration of ascorbic acid, l/s, and process temperature. The stages of the study were conducted to optimize the leaching parameters to achieve the highest leaching efficiency of lead from flash smelting slag. Through this approach, a lead leaching efficiency exceeding 61% was achieved with the following process parameters: ascorbic acid concentration of 0.75 mol/dm^3^, l/s = 40, temperature of 50 °C, and process time of 60 min. Under these conditions, high leaching efficiency for lead (>60%) and low leaching efficiencies for copper (~0.2%) and iron (~3.7%) were obtained. Such leaching efficiencies of the three analyzed elements indicate the high selectivity of ascorbic acid under the imposed conditions.

In the next series of experiments, hydrogen peroxide (30% H_2_O_2_) was added to the leaching solution of ascorbic acid to increase the leaching efficiency of lead. Two leaching tests were conducted with optimized process parameters obtained in the previous series and two additions of hydrogen peroxide (10 and 20 mL) as well as two tests with increased ascorbic acid concentrations (1 and 1.5 mol/dm^3^) and the addition of 10 mL of hydrogen peroxide. In each measurement, an increase in the leaching efficiency of all the analyzed metals was observed. Under the conditions of leaching slag with ascorbic acid solution at a concentration of 0.75 mol/dm^3^ with the addition of 10 mL of hydrogen peroxide, the highest lead leaching efficiency of 77% was achieved, whereas the leaching efficiencies of copper and iron were 5% and 5.2%, respectively.

Leaching of flash smelting slag with a mixture of ascorbic and citric acids in the presence of an oxidizer resulted in the achievement of a lead leaching efficiency of 88.1%. The leaching efficiencies of copper and iron under these conditions were 29% and 12%, respectively. Notably, after 60 min of the process, 87% of Pb, 23% of Cu, and 9% of Fe were transferred from the slag to the solution. Shortening of the process resulted in a slightly lower lead leaching efficiency and considerably lower efficiencies for copper and iron—the selectivity of leaching after 60 min was higher compared with the result obtained after 120 min of leaching.

The analysis of solid and liquid products obtained after leaching flash smelting slag with a solution of ascorbic acid in the presence of an oxidizing agent (variant “A”) as well as a mixture of ascorbic and citric acids in the presence of an oxidizing agent (variant “B”) confirmed the high efficiency of the applied solutions in leaching lead from the solid to the liquid phase. The EDS analysis revealed that the efficiencies were approximately 92% and almost 100% for variants “A” and “B”, respectively. Performance calculations based on the results of the ICP-OES analysis of leaching solutions indicated values above 79% for variant “A” and almost 92% for variant “B”. Therefore, it can be said that the conducted studies in this work led to achieving parameters of the leaching process where approximately 80% to approximately 90% of the lead contained in the initial slag sample could be transferred to the solution.

Analysis of the behavior of copper and iron during the conducted process led to the conclusion that both these elements transfer to the solution to a much lesser extent than lead. When using ascorbic acid in the presence of an oxidizing agent, the copper leaching efficiency ranged from 4% (according to the ICP-OES analysis) to 5% (according to the MP-AES analysis) depending on the analytical method used. The efficiencies for iron were 5.2% (MP-AES) and 6.6% (ICP-OES). When using a mixture of ascorbic and citric acids in the presence of an oxidizing agent, considerably higher leaching efficiencies for copper and iron were obtained compared with the previous attempt. For Cu, the leaching efficiencies were 29% (MP-AES) and 32.4% (ICP-OES), whereas for iron, they were 12% (MP-AES) and 12.6% (ICP-OES). Therefore, it can be concluded that the use of a mixture of ascorbic and citric acids instead of only ascorbic acid increases the lead leaching efficiency and decreases the selectivity of this process considering the other two metals analyzed in the study.

Comparing the obtained results with the results of research on the use of acetic acid [30] for leaching lead from flash smelting slag, it can be observed that the use of ascorbic acid solutions led to higher leaching efficiencies for lead but lower leaching efficiencies for copper. In the case of ascorbic acid solutions with perhydrol, with optimized process parameters, the leaching efficiencies were 77% and 5%, respectively. When using acetic acid solutions, leaching efficiencies in the range of 60–65% for lead and 21–61% for copper were obtained. The use of ascorbic acid in the leaching process proceeds with greater selectivity compared with the use of acetic acid and allows for the removal of lead from slag with considerably higher efficiency. In the case of leaching slag using citric acid [29], approximately 85% to 90% of the lead contained in the initial slag sample and between 25% and 32% of the copper were passed into the solution. The efficiency for lead was slightly higher than those obtained when using ascorbic acid with H_2_O_2_. Hence, the use of a mixture of two acids and perhydrol in the leaching process led to an increase in the extraction of lead (~88%) and copper (~29%) into the solution to levels similar to those obtained using citric acid.

Looking at the results from the perspective of the potential industrial application of the proposed process, the experiments using ascorbic acid with the addition of hydrogen peroxide appear promising. The use of an l-ascorbic acid solution with a concentration of 1.5 mol/dm^3^ and 10 mL of H_2_O_2_ resulted in the transfer of 68% of lead and 4% of iron into the liquid phase after 30 min. However, no copper transfer to the solution was observed. It can be assumed that after such a process, the lead content in the slag may decrease to approximately 0.95%, and after subsequent decopperization to about 1.1–1.2%. This level is slightly lower than that achieved in the prolonged, high-temperature, and energy-intensive process of decopperization of flash smelting slag conducted in an electric furnace, which also entails significant CO_2_ emissions. The hydrometallurgical process could induce copper and lead recovery at a similar level without the aforementioned negative consequences.

## 4. Experimental

In this study, flash smelting slag from Głogów Copper Smelter II (KGHM, Lubin, Poland) was used for leaching experiments. Results of the chemical analysis of this slag conducted using the flame atomic absorption spectrometry (FAAS) (AAS6000, Skyray Instrument, Dallas, TX, USA) method (performed in the laboratory of Głogów Copper Smelter) are presented in Table 10. The examined slag is a fine-grained material that does not form agglomerates. After grinding, the slag was sieved, and the fraction above 0.5 mm was reground to ensure that the entire volume of slag after comminution did not exceed a grain diameter of 0.5 mm.

**Figure 18:**
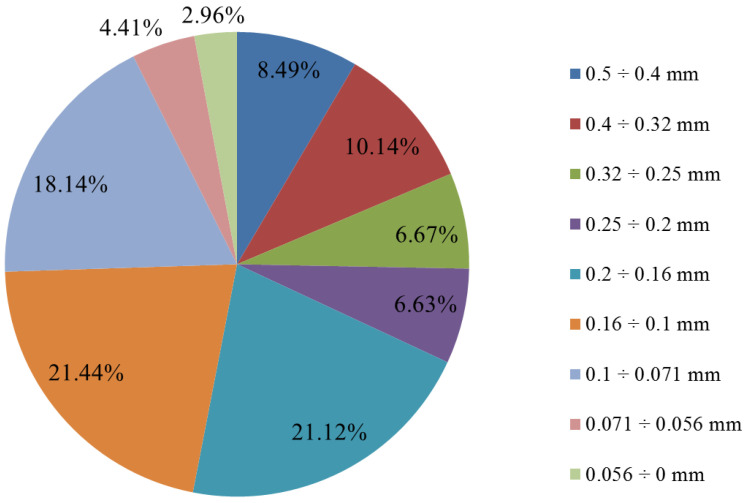
Results of the sieve analysis of flash smelting slag.

The analyzed slag had a grain size of below 0.5 mm. However, to more precisely determine the share of individual grain classes, a sieving test was conducted using 9 sieves with different mesh sizes. As can be seen from Figure 18, the examined slag does not contain a prominently distinguishable grain class. The two finest grain classes (below 0.056 mm, and 0.056–0.071 mm) each account for less than 5% of mass fraction. The highest shares are characterized by the classes 0.071–0.1 mm (18.14%), 0.1–0.16 mm (21.44%), and 0.16–0.2 mm (21%). The remaining four fractions with the largest grains range between 6.6% and 10.2% in their shares.

**Figure 19:**
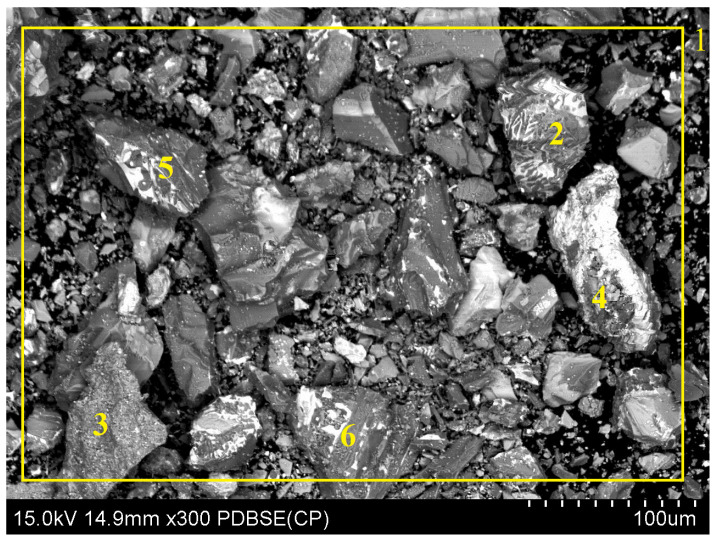
SEM analysis of the slag sample along with the chemical composition analysis of the observed field (1) and characteristic points (2–6).

The slag intended for analysis was observed and assessed through scanning electron microscopy (SEM) (S-4800, Hitachi, Tokyo, Japan). The results of the present study, in the form of an image of the sample along with the analysis of the chemical composition of the observed field, as well as the selected characteristic points of the sample, are shown in Figure 19 and Table 11. The conducted research reported significant variations in the chemical composition of slag grains comprising the analyzed sample. The average contents of copper, lead, and iron determined through SEM are close to the values determined using the FAAS method. However, due to the nonrepresentative nature of SEM analysis, all the results obtained in this study were supplemented with results obtained from the FAAS analysis.

**Figure 20:**
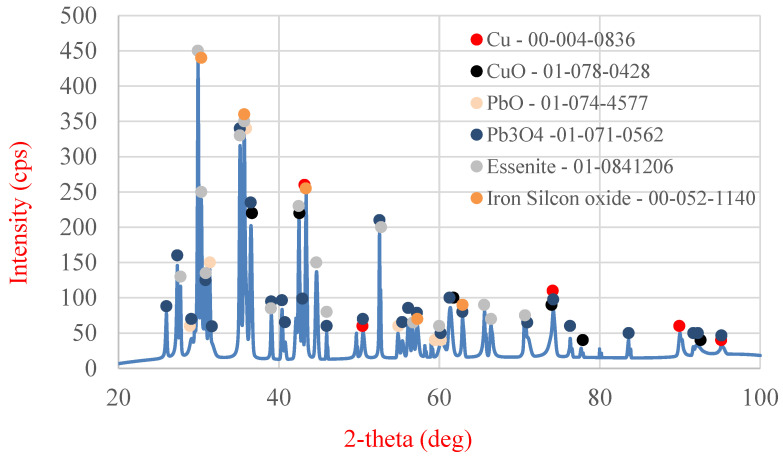
XRD analysis of the initial slag sample.

The final stage of the analysis of the starting material was X-ray diffraction (XRD) examination (MiniFlex II, Rigaku, Tokyo, Japan) (Figure 20), enabling the determination of the phase composition of the slag. The following phases were identified in the analyzed slag sample: metallic copper, copper oxide (II), lead oxide (II), lead tetroxide (Pb_3_O_4_), essenite (CaFeAlSiO_6_), and a complex oxide containing iron and silicon (Fe_2.95_Si_0.05_O_4_).

**Figure 21:**
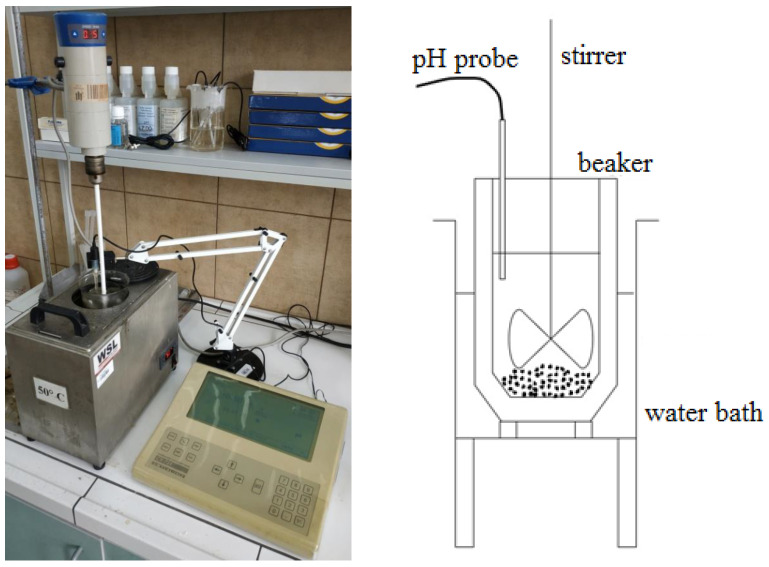
Measurement setup for the leaching tests of flash smelting slag.

The leaching tests were conducted in a batch reactor with external heating, equipped with a stirrer and a pH probe for monitoring the pH value of the solution. The measurement setup is depicted in Figure 21. The reactor used for the process was a 600-mL beaker, and a 400-mL solution was utilized in all the trials. The following leaching reaction profiles for lead (1), copper (2), and iron (3) were assumed using ascorbic acid:

(1) $${2C}_{6}H_{8}O_{6}+PbO\to C_{12}H_{14}{PbO}_{12}+H_{2}O$$

(2) $${2C}_{6}H_{8}O_{6}+CuO\to C_{12}H_{14}CuO_{12}+H_{2}O$$

(3) $${2C}_{6}H_{8}O_{6}+FeO\to C_{12}H_{14}{FeO}_{12}+H_{2}O$$

The aim of the conducted analyses was to examine the leaching process of Pb, Cu, and Fe over time. Thus, samples were taken for analysis during the measurement. For each measurement, a fresh l-ascorbic acid (99% Sigma-Aldrich, St. Louis, MO, USA) solution at an appropriate concentration was prepared. Before starting the measurement, the solution was heated to the required temperature. When the measurement system was ready, the mechanical stirrer was switched on at a speed of 250 rpm. The test was initiated with the addition of the slag sample to the solution of acid. In some trials, perhydrol (30% Sigma-Aldrich) was added in four portions: at the beginning of the study and 30, 60, and 90 min after the start of the experiment. Solution samples with a volume of 3.5 cm^3^ were taken using a pipette at appropriate time intervals, i.e., after 5, 10, 20, 30, 60, 90, and 120 min. The collected samples were then filtered, separating the solution for analysis from a small amount of sediment drawn during sampling. Finally, the obtained filtrate was collected using an automatic pipette with a capacity of 1 or 0.5 cm^3^ (depending on the expected element concentrations) and poured into 50 cm^3^ volumetric flasks filled with 5% nitric acid. Dilutions were used to prevent the precipitation of sediments along with a decrease in temperature.

The diluted samples were mixed, adjusted to 50 cm^3^, and then analyzed using an MP-AES spectrometer (Agilent MP-AES 4200, Agilent Technologies, Santa Clara, CA, USA). To examine the content of the elements of interest, standards containing appropriate concentrations of the elements were prepared beforehand. The leaching efficiency was determined as follows:

(4) $$\eta_{i}=\left(\frac{m_{i}}{m_{i}^{0}}\right)$$

where

$m_{i}^{0}$—mass of the element “i” in the slag sample before the process

$m_{i}$—mass of the element “i” in the solution after the leaching process

*i* = Pb, Cu, Fe

In addition to the main analyses conducted for all samples, certain specified solutions and solid residues after leaching were further examined. These investigations included the sediment after leaching with the highest lead leaching efficiency (SEM and XRD) as well as the solution from this sample (ICP-OES instrument, Optima 7300DV, Perkin Elmer, Waltham, MA, USA).

## 5. Conclusions

Hydrometallurgical processing of nonferrous metal industrial waste may present an interesting alternative to currently employed pyrometallurgical technologies. The use of solutions of inorganic acids such as acetic, citric, and ascorbic acids for this purpose can be particularly appealing owing to their minimal impact on the natural environment and the relatively low cost of reagents. The leaching studies of flash smelting slag using ascorbic acid solutions conducted in this work led to the following conclusions:

1. Leaching of slag using solutions of ascorbic acid mainly results in the dissolution of lead into the solution, along with minor amounts of copper and iron.
2. The parameters significantly affecting the aforementioned process include the l/s ratio, ascorbic acid concentration, and leaching time, whereas only a minor influence of temperature on the leaching efficiency of lead is observed.
3. The addition of an oxidizing agent in the form of H_2_O_2_ increases the leaching efficiencies of lead, copper, and iron, albeit at the expense of process selectivity.
4. Process parameter optimization allows for selective leaching of lead with an efficiency of 68% (0% Cu, 4% Fe).
5. The use of a mixture of ascorbic acid, citric acid, and hydrogen peroxide in the process results in leaching efficiencies of approximately 88% for lead, 29% for copper, and 12% for iron.

## Figures and Tables

**Table 1:** pH values of the solutions measured during the leaching tests conducted for various concentrations of l-ascorbic acid.

	pH		
t [min]	0.25 mol/dm^3^	0.50 mol/dm^3^	0.75 mol/dm^3^
0	1.76	1.59	1.46
5	2.97	2.72	2.58
10	3.07	2.81	2.66
20	3.16	2.87	2.72
30	3.22	2.91	2.75
60	3.32	2.95	2.78
90	3.39	2.98	2.81
120	3.44	3.00	2.82

**Table 2:** pH values of the solutions measured during the experiments conducted for different l/s parameters.

	10	20	40
t [min]	pH	pH	pH
0	1.46	1.48	1.48
5	2.58	2.37	2.15
10	2.66	2.44	2.21
20	2.72	2.48	2.24
30	2.75	2.51	2.27
60	2.78	2.51	2.28
90	2.81	2.52	2.28
120	2.82	2.52	2.27

**Table 3:** pH values of the solutions measured during the experiments conducted at different temperatures.

	50 °C	70 °C	90 °C
t [min]	pH	pH	pH
0	1.48	1.20	1.01
5	2.15	1.94	1.74
10	2.21	1.99	1.79
20	2.24	2.01	1.80
30	2.27	2.00	1.80
60	2.28	2.03	1.80
90	2.28	2.04	1.80
120	2.27	1.97	1.79

**Table 4:** pH values of the solutions measured during leaching with l-ascorbic acid with the addition of hydrogen peroxide.

	0.75 mol/dm^3^		1 mol/dm^3^	1.5 mol/dm^3^
	10 mL	20 mL	10 mL	
t [min]	pH	pH	pH	pH
0	1.43	1.43	1.29	1.08
5	2.11	2.06	1.91	1.72
10	2.17	2.10	1.99	1.79
20	2.20	2.17	2.00	1.82
30	2.20	2.14	2.03	1.81
60	2.14	1.93	1.98	1.74
90	2.00	1.70	1.89	1.63
120	1.87	1.51	1.75	1.53

**Table 5:** pH values of the solution for the tests with the mixture of ascorbic and citric acids and the addition of hydrogen peroxide.

t [min]	pH
0	0.62
5	0.93
10	1.00
20	1.01
30	1.07
60	1.03
90	1.04
120	1.01

**Table 6:** Elemental content in the solid phase after leaching using ascorbic acid in the presence of an oxidizing agent (EDS analysis, weight, %).

	O	Mg	Al	Si	Ca	Fe	Cu	Zn	Pb
**area1**	36	3	4	17	10	15	14	1	0
**2**	3	0	1	2	1	78	6	8	0
**3**	6	1	2	8	36	42	4	0	0
**4**	31	4	4	1	0	50	3	7	0
**5**	4	0	1	2	1	46	42	3	0
**6**	47	6	5	18	14	9	1	0	0

**Table 7:** ICP-OES analysis of the leachate after leaching using ascorbic acid in the presence of an oxidizing agent.

Element	Pb	Cu	Fe	Ag	Al	As	B	Ba
g/dm^3^	**0.591**	**0.136**	**0.193**	0.001	0.295	0.025	0.033	0.008
Ca	Cd	Co	Cr	K	Li	Mg	Mn	Mo
0.557	0.001	0.003	0.001	0.301	0.001	0.031	0.019	0.015
Na	Ni	S	Si	Sr	V	Zn	P	
0.317	0.001	0.100	0.309	0.006	0.018	0.124	0.050	

**Table 8:** Elemental content in the solid phase after leaching using a mixture of ascorbic acid and citric acid in the presence of an oxidizing agent.

	O	Mg	Al	Si	Ca	Fe	Cu	Zn	Pb
**area1**	37	4	4	16	11	19	9	1	0
**2**	28	3	4	11	5	32	16	1	0
**3**	30	2	3	14	26	22	1	2	0
**4**	50	2	3	28	10	8	1	0	0
**5**	49	0	0	43	0	1	7	0	0
**6**	3	0	1	1	0	2	93	0	0

**Table 9:** ICP-OES analysis of the solution after leaching using a mixture of ascorbic acid and citric acid in the presence of an oxidizing agent.

Element	Pb	Cu	Fe	Ag	Al	As	B	Ba
g/dm^3^	**0.675**	**1.091**	**0.368**	0.001	0.449	0.028	0.024	0.012
Ca	Cd	Co	Cr	K	Li	Mg	Mn	Mo
0.600	0.001	0.004	0.003	0.495	0.001	0.040	0.022	0.017
Na	Ni	P	S	Si	Sr	V	Zn	
0.307	0.004	0.050	0.100	0.356	0.006	0.020	0.137	

**Table 10:** Content of basic elements in flash smelting slag used for research.

Element	Cu	Fe	Pb	Si	Ca	Al	Mg	Zn
Content [%]	13.48	11.67	2.98	14.43	8.93	4.21	3.06	0.96

**Table 11:** EDS analysis of flash smelting slag in selected points (weight, %).

	O	Mg	Al	Si	Ca	Fe	Cu	Zn	Pb
**area1**	37	3	5	15	11	13	12	2	4
**2**	44	0	13	31	4	3	1	0	5
**3**	33	2	5	15	18	14	8	3	4
**4**	11	0	1	1	0	1	86	0	0
**5**	25	3	4	18	20	16	8	2	4
**6**	53	5	5	17	12	7	0	1	0

## Data Availability

The data presented in this study are available on request from the corresponding author.
